# *De novo* Transcriptome Analysis and Molecular Marker Development of Two *Hemarthria* Species

**DOI:** 10.3389/fpls.2016.00496

**Published:** 2016-04-18

**Authors:** Xiu Huang, Hai-Dong Yan, Xin-Quan Zhang, Jian Zhang, Taylor P. Frazier, De-Jun Huang, Lu Lu, Lin-Kai Huang, Wei Liu, Yan Peng, Xiao Ma, Yan-Hong Yan

**Affiliations:** ^1^Department of Grassland Science, Animal Science and Technology College, Sichuan Agricultural UniversityChengdu, China; ^2^Herbivorous Livestock Research Institute, Chongqing Academy of Animal SciencesChongqing, China; ^3^Department of Horticulture, Virginia Polytechnic Institute and State UniversityBlacksburg, VA, USA; ^4^Department of Biochemistry and Molecular Biology, Biosciences Faculty, Universitat Autònoma de BarcelonaCerdanyola del Vallès, Spain

**Keywords:** *de novo* assembly, marker development, *Hemarthria* R. Br., RNA-Seq, transcriptome

## Abstract

*Hemarthria* R. Br. is an important genus of perennial forage grasses that is widely used in subtropical and tropical regions. *Hemarthria* grasses have made remarkable contributions to the development of animal husbandry and agro-ecosystem maintenance; however, there is currently a lack of comprehensive genomic data available for these species. In this study, we used Illumina high-throughput deep sequencing to characterize of two agriculturally important *Hemarthria* materials, *H. compressa* “Yaan” and *H. altissima* “1110.” Sequencing runs that used each of four normalized RNA samples from the leaves or roots of the two materials yielded more than 24 million high-quality reads. After *de novo* assembly, 137,142 and 77,150 unigenes were obtained for “Yaan” and “1110,” respectively. In addition, a total of 86,731 “Yaan” and 48,645 “1110” unigenes were successfully annotated. After consolidating the unigenes for both materials, 42,646 high-quality SNPs were identified in 10,880 unigenes and 10,888 SSRs were identified in 8330 unigenes. To validate the identified markers, high quality PCR primers were designed for both SNPs and SSRs. We randomly tested 16 of the SNP primers and 54 of the SSR primers and found that the majority of these primers successfully amplified the desired PCR product. In addition, high cross-species transferability (61.11–87.04%) of SSR markers was achieved for four other Poaceae species. The amount of RNA sequencing data that was generated for these two *Hemarthria* species greatly increases the amount of genomic information available for *Hemarthria* and the SSR and SNP markers identified in this study will facilitate further advancements in genetic and molecular studies of the *Hemarthria* genus.

## Introduction

*Hemarthria* R. Br. is one of the most important genera of the Poaceae family. This genus consists of about 20 species that are distributed across wide geographic regions. Among these species, the two most important and widely studied are *Hemarthria compressa* and *Hemarthria altissima*. *H. compressa*, also known as whipgrass, is mainly distributed in China and is one of the most significant and widely utilized forage grasses in southern China. Three commercial hexaploid (2n = 6x = 54) *H. compressa* cultivars (“Yaan,” “Guangyi” and “Chonggao”) have been released by the Sichuan Agricultural University (Yang et al., 2003; Guo et al., 2014). *H. altissima*, commonly known as limpograss, is native to Africa; however, it was later introduced into the southeastern part of the United States where its germplasms has since been evaluated. Four commercial *H. altissima* cultivars, including the diploids (2n = 2x = 18) “Greenalta” and “Redalta” and the tetraploids (2n = 4x = 36) “Bigalta” and “Floralta,” have been released (Huang et al., 2014d). These two species of *Hemarthria* have been extensively used and commercially grown in subtropical and tropical areas due to their fast growth, high forage yield, good quality, and tolerance to poorly drained soils. In addition, these two *Hemarthria* species are known to play an essential role in local animal husbandry and agro-ecosystem maintenance (Rumball and Lambert, 1981; Yang et al., 2003; Huang et al., 2014a,d).

Previous molecular studies on *Hemarthria* concentrated on estimating genetic diversity within naturally occurring germplasm, constructing DNA fingerprints, and associating molecular markers with agronomically important traits (Chen et al., 2011a; Guo et al., 2014; Huang et al., 2014a). A variety of molecular markers have been developed for *Hemarthria*, particularly for *H. compressa* and *H. altissima*, based on anonymous DNA sequences (Chen et al., 2005, 2011b; Liu et al., 2006; Huang et al., 2008, 2014b,c). In addition, only 19 PopSet (sequence sets from phylogenetic and population studies) and 37 nucleotide sequences of *Hemarthria* have been deposited in GenBank. *Hemarthria* species are some of the most economically and ecologically important forage crops and have been used for grazing, modulating hay, making silage, soil and water conservation, and greening the environment. Despite its singnificance, there is a serious lack of available genomic information available for *Hemarthria* spieces, which has greatly limited progress with breeding studies in this genus. Therefore, additional high quality genomic resources of *Hemarthria* are urgently needed in order to understand the molecular mechanisms underlying desirable agronomic traits in this important forage species.

High-throughput next-generation sequencing (NGS) technologies are rapidly transforming the fields of ecology, evolution, and genetics by increasing the amount of large-scale genomic and transcriptomic data that is available for non-model plant species (Rokas and Abbot, 2009; Renaut et al., 2010). NGS technologies can efficiently produce enormous amounts of sequence data at a significantly reduced cost. There are three main commercial NGS platforms including the Applied Biosystems SOliD System, Roche/454 GS FLX Instrument, and the Illumina/Solexa Genome Analyzer that can be widely applied to generate massively parallel DNA sequencing reads. Among these technologies, the Illumina sequencing platform is a short-read based technology that utilizes reversible terminator chemistry (Trick et al., 2009). Though the Illumina platform produces shorter reads than the other platforms, it offers several advantages over the others including a high level of accuracy, throughput, and cost effectiveness. In addition, a plethora of powerful bioinformatics tools have been developed for the analysis of Illumina sequencing data, which have addressed the short-comings of the sequencing technology. Currently, this sequencing-by-synthesis platform has been successfully used in whole genome sequencing (Li et al., 2010, 2013), digital gene expression analysis (Nagalakshmi et al., 2008), DNA-protein interaction profiling (Johnson et al., 2007), small RNA identification (Vidal et al., 2013), and the sequencing of transcriptomes (Sablok et al., 2014; Yates et al., 2014; Xie et al., 2015).

With the development of NGS technologies, RNA sequencing (RNA-Seq) has emerged as a powerful tool for transcriptome analysis. RNA sequencing can largely and precisely quantify gene expression levels with a high degree of sensitivity. Additionally, RNA-Seq has a high level of reproducibility for both technical and biological replicates. With minimal RNA input, RNA-Seq can also accelerate the assembly of transcriptomes and the identification of expressed genes including gene isoforms and alternatively spliced gene products (Cloonan et al., 2008; Nagalakshmi et al., 2008; Wang et al., 2009, 2010; Zhang et al., 2010; Vijay et al., 2013). RNA-Seq technology can extensively and accurately sequence expressed genes. It can also detect and characterize nucleotide variations such as SNPs and SSRs (Kaur et al., 2012). Finally, RNA-Seq does not rely on existing genomic sequences, which makes RNA-Seq especially useful for the analysis of non-model species that possess large nuclear genomes such as polyploids (Wang et al., 2009). To date, numerous RNA-Seq analyses have been reported for non-model plant species (Toledo-Silva et al., 2013; Wang et al., 2013; Yates et al., 2014); however, no RNA-Seq experiments have been performed for any *Hemarthria* species.

In this study, we used the Illumina HiSeq™2500 platform to perform a large-scale transcriptome analysis of two different *Hemarthria* species, *H. compressa* “Yaan” and *H. altissima* “1110.” *H. compressa* “Yaan” is one of three registered cultivars of *H. compressa* and is tall, light green in color, and has a high yield. *H. altissima* “1110,” a wild material of *H. altissima* that is derived from KwaZulu-Nata, South Africa, is also tall and high-producing. The main objectives of this study were to enrich the genomic resources available for *Hemarthria* species and to develop, characterize, and validate SNP and SSR molecular markers for the two materials. The results of this study will provide valuable genomic resources that contribute to (1) understanding the relationships among germplasm within the *Hemarthria* genus, (2) identifing *Hemarthria* varieties, (3) associating molecular markers with agronomically important traits of *H. compressa* and *H. altissima*, and (4) facilitating further advancements in marker-assisted selection (MAS), comparative transcriptomic studies, and candidate genes research in *Hemarthria* species.

## Materials and methods

### Ethics statement

This study was approved by the Department of Grassland Science, Animal Science and Technology College, Sichuan Agricultural University. *H. compressa* “Yaan” and *H. altissima* “1110” are not endangered or protected materials.

### Plant material and RNA isolation

*Hemarthria* species are infertile grasses that propagate through rhizomes. In this study, the rhizomes of *H. compressa* “Yaan” and *H. altissima* “1110” were used for further research. Rhizomes of each material were collected from individual clones and were cultivated in Erlenmeyer flasks (200 mL volume) containing Hoagland's nutrient solution (Hoagland and Arnon, 1950). The Erlenmeyer flasks containing the rhizomes were placed in a temperature-controlled growth chamber programmed at 28°C/19°C (average day/night temperature) under a light/dark photoperiod of 16 h/8 h. The nutrient solution was changed every 3 days. After 2 months of growth, the leaves and roots of two individuals per material were collected separately and immediately frozen in liquid nitrogen. The tissue was stored at −80°C until RNA isolation.

To isolate RNA, equal amounts of leaf or root tissue from individual clones of the same material were pooled together to create one sample. The samples were labeled as follows: T01 = the mixed leaves of “Yaan,” T02 = the mixed roots of “Yaan,” T03 = the mixed leaves of “1110,” and T04 = the mixed roots of “1110.” Total RNA was extracted using an RNAprep pure Plant Kit (Tiangen Biotech, China) in accordance with the manufacturer's protocol. The purity and concentration of total RNA was detected using a Nanodrop 2000 UV-Vis spectrophotometer (Thermo Fisher Scientific Inc., USA) and a Qubit® 2.0 Fluorometer (Life Technologies, USA). The RNA Integrity Number (RIN) for the four RNA samples was examined with an Agilent 2100 Bioanalyzer (Agilent Technologies, USA).

### cDNA library construction and illumina sequencing

The four total RNA samples from the two *Hemarthria* species were sent to Biomarker Technologies Co., Ltd. (Beijing, China) for the construction of cDNA libraries and for Illumina sequencing reactions. After the total RNA was extracted, the mRNA in each of the samples was purified and enriched using magnetic oligo(dT)-rich beads. Next, a specialized buffer and high temperatures were used to chemically break the mRNA into fragments. Using reverse transcriptase and random hexamer-primers, the cleaved mRNA fragments were then used as templates to synthesize first-strand cDNA. Next, second-strand cDNA was synthesized in a buffer containing dNTPs, DNA polymerase I, and RNaseH. After purification of the double-stranded cDNA with Agencourt AMPure XP beads, the purified cDNA ends were repaired using T4 DNA polymerase and Klenow DNA polymerase, which adds a single A base to the end of the sequence. The repaired cDNA fragments were then ligated to sequencing adapters. Agencourt AMPure XP beads were used to select suitable length fragments that functioned as sequencing templates for downstream analyses. Next, PCR amplification was then performed using the Phusion High-Fidelity DNA polymerase in order to enrich the purified cDNA template. To ensure that the quality of the library was sufficient for sequencing, the concentration and insert size of the library was detected using a Qubit® 2.0 Fluorometer and Agilent 2100 Bioanalyzer, respectively. Additionally, the effective concentration of the library was accurately quantified using quantitative PCR (Q-PCR). Finally, the qualified cDNA library was sequenced on the Illumina HiSeq™ 2500 sequencing platform using paired-end technology in a single run.

### *De novo* transcriptome assembly and annotation

The abundant raw reads that were obtained from the Illumina sequencing platform (NCBI SRA: SRP058845) were initially processed in order to ensure the accuracy of *de novo* assembly and subsequent analyses. The pre-processing guidelines included trimming reads with adaptors and eliminating low quality reads (reads with ambiguous “N” bases >5% and more than 10% Q < 20 bases). The clean reads from each library were evaluated for GC-content, N-content, Q20, CycleQ20, and Q30. Next, Trinity (http://trinityrnaseq.sourceforge.net/) software, which is specific for high-throughput transcript assembly of RNA-Seq data without a reference genome (Grabherr et al., 2011), was used to separately assemble the clean reads from each library into unigene sequences with the parameters set at K-mer length of 25, a similarity of 80%, and the other parameters set to their default values. The Trinity software used three independent modules: Inchworm, Chrysalis and Butterfly, which first combined all the clean reads with a certain length of overlap to form contigs (longer contiguous fragments without N). Then, the software let the clean reads map back to the contigs and used paired end reads to calculate the distance and relation among these contigs. Next, Trinity connected these contigs to obtain consensus sequences that could not be extended on either end were called transcripts. Finally, the transcripts were then further clustered into unigenes.

The program BLAST (http://blast.ncbi.nlm.nih.gov/Blast.cgi) was used to assign putative functions to the assembled unigenes (Altschul et al., 1997). All of the unigene sequences were aligned using BLASTx (*e* < 1e-5) to the following publicly available protein databases: National Center for Biotechnology Information (NCBI) non-redundant protein (Nr), Swiss-Prot protein, Protein family (Pfam), Eukaryotic Orthologous Groups of proteins (KOG), Gene Ontology (GO), and the Kyoto Encyclopedia of Genes and Genomes (KEGG). In addition, a BLASTn search was performed against the NCBI non-redundant nucleotide sequence (Nt) database with a cut-off *E*-value of 10^−5^. Next, functions were assigned to the unigenes with priority given to the seven databases in the following order: Nr, GO, KEGG, Swiss-Prot, Pfam, KOG, and Nt (Moriya et al., 2007; Finn et al., 2008) The best BLAST alignments from this study were selected in order to determine the sequence direction of the unigenes, as well as to predict the coding regions (CDSs). Next, the CDS regions of the unigenes were translated into their protein sequences according to standard genetic codes. For the unigenes that did not align to any of the above databases, the Getorf program (http://emboss.sourceforge.net/apps/cvs/emboss/apps/getorf.html) was used to predict their open reading frames (ORFs). The longest open-ended ORF was extracted as the most probable translated region for each unigene.

### Identification and validation of SNPs

To identify SNPs in *H. compressa* and *H. altissima* that could be developed as molecular markers, the sequencing data from “Yaan” and “1110” were compared to the unigene database using SOAPsnp software (http://soap.genomics.org.cn/soapsnp; Li et al., 2009). The thresholds for SNP identification were as follows: the prior probability values for novel heterozygous SNPs and the prior probability values for novel homozygous SNPs were set at 0.0001 and 0.0005, respectively; the sequencing depth was between 10X and 100X; and the consensus base quality score had to be ≥30 (quality score 30 represents 99.9% accuracy of a base call).

Twenty *Hemarthria* materials were used to validate a subset of the SNP markers identified in this study (Table S2). The same amount of leaf tissue was harvested for each accession and DNA was extracted using a genomic DNA extraction kit (Tiangen Biotech, China) following the manufacturer's protocol. SNP primers were randomly selected from primer sets that were generated in our study (Table S3). PCR amplification reactions were performed in 30 μL volumes and contained 2 μL (20 ng/μL) of DNA template, 15 μL Premix *Taq* (TakaRa *Taq* Version 2.0 plus dye; TakaRa Bio Inc., China), 2 μL (10 pmol/μL) each of the forward and reverse primers, and 9 μL ddH_2_O. The PCR cycling conditions for SNP detection were as follows: 5 min pre-denaturation at 94°C, followed by 35 cycles of 1 min denaturation at 94°C, 1 min annealing at 53°C, 1 min extension at 72°C, and a final 10 min extension at 72°C. PCR products were separated on 1.5% agarose gels at 130 V for 30 min and then visualized using UV light. The PCR products that were the correct size were then purified by gel extraction and sent to the Beijing Genomics Institute for gene sequencing.

### Identification, validation, and cross-species transferability of SSRs

The assembled sequences of all four data sets that were longer than 1 kb were merged for microsatellite mining using MISA software (http://pgrc.ipk-gatersleben.de/misa/). The sequences were searched for perfect mono-, di-, tri-, tetra-, penta-, and hexa-nucleotide motifs with a minimum of 10, six, five, five, five, and five repeats, respectively. We also searched for compound SSRs with a minimum distance of less than 100 nt between two single SSRs. Based on the results from the MISA software, Primer 3 v 2.23 (http://primer3.sourceforge.net) was used to design PCR primers in the flanking regions of the SSRs. To acquire a high probability of amplification, the primer design parameters were set as follows: the primer length range was 18–23 nt, with the optimal length at 21 nt; the PCR products were between 100 and 300 bp long; the annealing temperature range was between 52 and 58°C, with 55°C as the optimum; and the GC percentage ranged from 40 to 60%, with an optimal GC content of 50%.

Forty-four *Hemarthria* materials were used to validate the SSR markers identified in this study (Table S2). In addition, we explored the transferability of the identified SSR makers to other four Poaceae genera (*Lolium, Pennisetum, Miscanthus*, and *Dactylis*) and analyzed their presence/absence in seven *Lolium multiflorum* cultivars, five *Pennisetum* varieties, four wild *Miscanthus sinensis* accessions, and six *Dactylis glomerata* cultivars (Table S5). The genomic DNA of these materials was extracted from young leaves using a genomic DNA extraction kit (Tiangen Biotech, China) in accordance with the manufacturer's protocol. To evaluate SSR polymorphic differences and transferability within the Poaceae family, we randomly chose 54 SSR primers from our list of self-designed SSR primers (the final selected SSR primer sequences for validation is shown in Table S4). For PCR amplification of the SSR primers, reactions were performed in 15 μL total volume and included 1.5 μL (20 ng/μL) of genomic DNA, 7.5 μL of 2X Reaction Mix (Tiangen, Biotech, China), 0.6 μL (10 pmol/μL) of each forward and reverse primer, 0.3 μL of Golden DNA Polymerase (Tiangen Biotech, China), and 4.5 μL of distilled water. The PCR amplification program included a pre-denaturation at 94°C for 5 min, followed by 35 cycles of 94°C for 30 s, 52–56°C for 45 s, and 72°C for 1 min with a final extension step at 72°C for 10 min. The obtained PCR products were examined using the method described by Huang et al. (2014d).

## Results and discussion

### Sequence analysis and assembly

Illumina RNA-Seq technology has the ability to generate billions of reads and has been successfully used for transcriptome sequencing and *de novo* transcriptome assembly. As such, RNA-Seq has emerged as an important tool for gene discovery and molecular marker development. In this study, we used the Illumina HiSeq™ 2500 platform to generated more than 24,528,820 raw reads (~4.95 Gb) for each of the four *Hemarthria* RNA samples. More than 24,240,920 high-quality reads (~4.89 Gb clean data) were obtained for each sample after quality control trimming and filtering. The GC content, reads with “N” proportion, and cycleQ20 proportion of the clean data was over 54.61, 0.04, and 100.00%, respectively, for each of the four samples. More than 91.90% of the cycles had an average Phred score greater than 20, and the average base quality value was 30 or greater (≥30) for more than 85.46% of the cycles (Table S1). These results reflect the quality of the clean data reads and demonstrate that the clean reads were sufficient for subsequent analysis.

Using the Trinity *de novo* assembly software, 9,660,044 and 7,707,533 contigs were obtained for “Yaan” and “1110,” respectively. Using clustering and local assembly analyses, the contigs from “Yaan” were assembled into 269,972 transcripts with a mean length of 892.91 nt, and the contigs from “1110” were assembled into 170,550 transcripts with an average size of 1059.80 nt. The other characteristics of the contigs are given in Table 1. For the cultivar “Yaan,” these 269,972 transcripts were then further clustered into 137,142 unigenes with a mean length of 566.31 nt and an N50 length of 826 bp. For *H. altissima* “1110,” the 170,550 transcripts were clustered into 77,150 unigenes with a mean length of 679.26 nt and an N50 length of 1189 bp. After *de novo* assembly, the total transcriptome size was estimated to be 74.07 Mb and 49.98 Mb for “Yaan” and “1110,” respectively. These results indicate that the two *Hemarthria* materials used have complex genomes. The larger transcriptome size of “Yaan” could be attributed to the larger genome size of *H. compressa* compared to *H. altissima*, which is consistent with previous reports (Yang et al., 2003; Guo et al., 2014). In “Yaan,” 62,639 or 45.67% of the unigenes were 200–300 nt in size followed by 23,145 unigenes (16.88%) that were 300–400 nt in size. Similarly, in the “1110” material 30,958 unigenes or 40.13% were 200–300 nt size, followed by 11,936 unigenes (15.47%) that were 300–400 nt long (Table 2). Interestingly, a high proportion of the unigenes (40–45%) assembled in this study were shorter than 300 nt, indicating that these transcripts were fragmented. This could be explained by the fact that the clean sequencing data of young leaves and roots of the same material were used in the assembly process. Additionally, the short unigenes could be attributed to insufficient sequencing depth (~4.95 Gb). Despite the high percentage, the proportion of short unigenes generated for the two *Hemarthria* materials was similar to those observed in other non-model organisms, such as *Camellia sinensis* (44.36% < 300 bp) and *Oenanthe javanica* (40.34% < 300 bp; Jiang et al., 2014; Wu et al., 2014).

**Table 1:** **Summary of the transcripts for the two *Hemarthria* materials: *H. compressa* “Yaan” and *H. altissima* “1110”**.

**Transcripts length**	**Total number (percentage)**	
	**“Yaan”**	**“1110”**
200–300	76,195 (28.22%)	38,384 (22.51%)
300–500	53,478 (19.81%)	28,688 (16.82%)
500–1000	55,885 (20.70%)	35,220 (20.65%)
1000–2000	55,309 (20.49%)	42,683 (25.03%)
2000+	29,105 (10.78%)	25,575 (15.00%)
Total number	269,972	170,550
Tota length (nt)	241,059,806	180,748,233
N50 length (bp)	1504	1716
Mean length (nt)	892.91	1059.80

**Table 2:** **Summary of unigenes for the two *Hemarthria* materials: *H. compressa* “Yaan” and *H. altissima* “1110”**.

**Unigene length**	**Total number (percentage)**	
	**“Yaan”**	**“1110”**
200–300	62,639 (45.67%)	30,958 (40.13%)
300–400	23,145 (16.88%)	11,936 (15.47%)
400–500	11,839 (8.63%)	6339 (8.22%)
500–600	7198 (5.25%)	4014 (5.20%)
600–700	4961 (3.62%)	2831 (3.67%)
700–800	3679 (2.68%)	2327 (3.02%)
800–900	2925 (2.13%)	1932 (2.50%)
900–1000	2441 (1.78%)	1661 (2.15%)
1000–1100	1965 (1.43%)	1435 (1.86%)
1100–1200	1706 (1.24%)	1333 (1.73%)
1200–1300	1535 (1.12%)	1198 (1.55%)
1300–1400	1327 (0.97%)	1066 (1.38%)
1400–1500	1256 (0.92%)	958 (1.24%)
1500–1600	1071 (0.78%)	958 (1.24%)
1600–1700	948 (0.69%)	880 (1.14%)
1700–1800	940 (0.69%)	759 (0.98%)
1800–1900	827 (0.60%)	723 (0.94%)
1900–2000	735 (0.54%)	640 (0.83%)
2000–2100	686 (0.50%)	582 (0.75%)
2100–2200	560 (0.41%)	475 (0.62%)
2200–2300	573 (0.42%)	477 (0.62%)
2300–2400	464 (0.34%)	411 (0.53%)
2400–2500	391 (0.29%)	404 (0.52%)
2500–2600	387 (0.28%)	318 (0.41%)
2600–2700	341 (0.25%)	294 (0.38%)
2700–2800	270 (0.20%)	266 (0.34%)
2800–2900	210 (0.15%)	234 (0.30%)
2900–3000	228 (0.17%)	185 (0.24%)
3000–3100	186 (0.14%)	147 (0.19%)
>3000	1709 (1.25%)	1409 (1.83%)
Total number	137,142	77,150
Tota length (nt)	77,665,144	52,404,585
N50 length (bp)	826	1189
Mean length (nt)	566.31	679.26

Although *Hemarthria* species are economically and ecologically important forage crops, there is a serious lack of available genomic information available for them. No EST database currently exists for *Hemarthria* species. In this study, we have obtained a large number of representative transcript sequences of *Hemarthria* genes using Illumina RNA-Seq technology. The sequencing reads provide valuable genomic information for *Hemarthria* spieces, which can be utilized not merely for gene discovery but also for molecular marker (SSRs and SNPs) identification.

### Sequence annotation

Functional annotation and classification of transcriptomes can shed light on intracellular metabolic pathways and biological behaviors of genes (Tang et al., 2014). To predict the potential functions of the assembled unigenes, the obtained unigene sequences for the two *Hemarthria* materials were aligned using BLAST (*e* < 1e-5) to various databases, including Nt, Nr, Swiss-Prot, Pfam, GO, KOG, and KEGG. Amongst the 137,142 unigenes obtained from “Yaan,” 58,661 (42.77%) had significant hits in the Nt database, 75,478 (55.04%) in Nr, 46,906 (34.20%) in Swiss-Prot, 44,285 (32.29%) in Pfam, 50,638 (36.92%) in GO, 42,047 (30.66%) in KOG, and 18,148 (13.23%) in KEGG. For the 77,150 unigenes assembled for “1110,” 40,408 (52.38%) were a significant match to Nt, 41,262 (53.48%) to Nr, 28,513 (36.96%) to Swiss-Prot, 25,190 (32.65%) to Pfam, 30,538 (39.58%) to GO, 21,867 (28.34%) to KOG, and 8434 (10.93%) to KEGG. Thus, the number of “1110” unigenes that were matched to each database was only about one-half of the number of unigenes that were matched to the databases for “Yaan.” In total, 86,731 unigenes (63.24%) and 48,645 unigenes (63.05%) were successfully annotated for “Yaan” and “1110,” respectively (Table 3). The ratios of annotated unigenes of the two *Hemarthria* materials analyzed in our study were comparable to the range of previously reported annotated unigenes in other non-model organisms (Li et al., 2012; Lu et al., 2012). The remaining unigenes (36.76% in “Yaan” and 36.95% in “1110”) did not align with any known genes. This could be attributed to either a large number (40–45%) of unigenes that had lengths shorter than 300 nt, or a lack of relevant genetic data. For both the unigenes identified in the “Yaan” and “1110,” the most frequent and significant annotation hits in the databases were to four well-annotated monocot plant species: *Sorghum bicolor, Zea mays, Setaria italic*, and *Oryza sativa* Japonica Group. These matched species all belong to the Poaceae family, supporting that the sequences obtained in our study were annotated properly.

**Table 3:** **Functional annotation of the *Hemarthria* transcriptome for *H. compressa* “Yaan” and *H. altissima* “1110”**.

**Databases**	**Total number (percentage)**	
	**“Yaan”**	**“1110”**
Annotated in Nt	58,661 (42.77%)	40,408 (52.38%)
Annotated in Nr	75,478 (55.04%)	41,262 (53.48%)
Annotated in Swiss-Prot	46,906 (34.20%)	28,513 (36.96%)
Annotated in Pfam	44,285 (32.29%)	25,190 (32.65%)
Annotated in GO	50,638 (36.92%)	30,538 (39.58%)
Annotated in KOG	42,047 (30.66%)	21,867 (28.34%)
Annotated in KEGG	18,148 (13.23%)	8434 (10.93%)
Annotated in all databases	7628 (5.56%)	4250 (5.51%)
Annotated in at least one databases	86,731 (63.24%)	48,645 (63.05%)
Total unigenes	137,142 (100.00%)	77,150 (100.00%)

**Figure 1:**
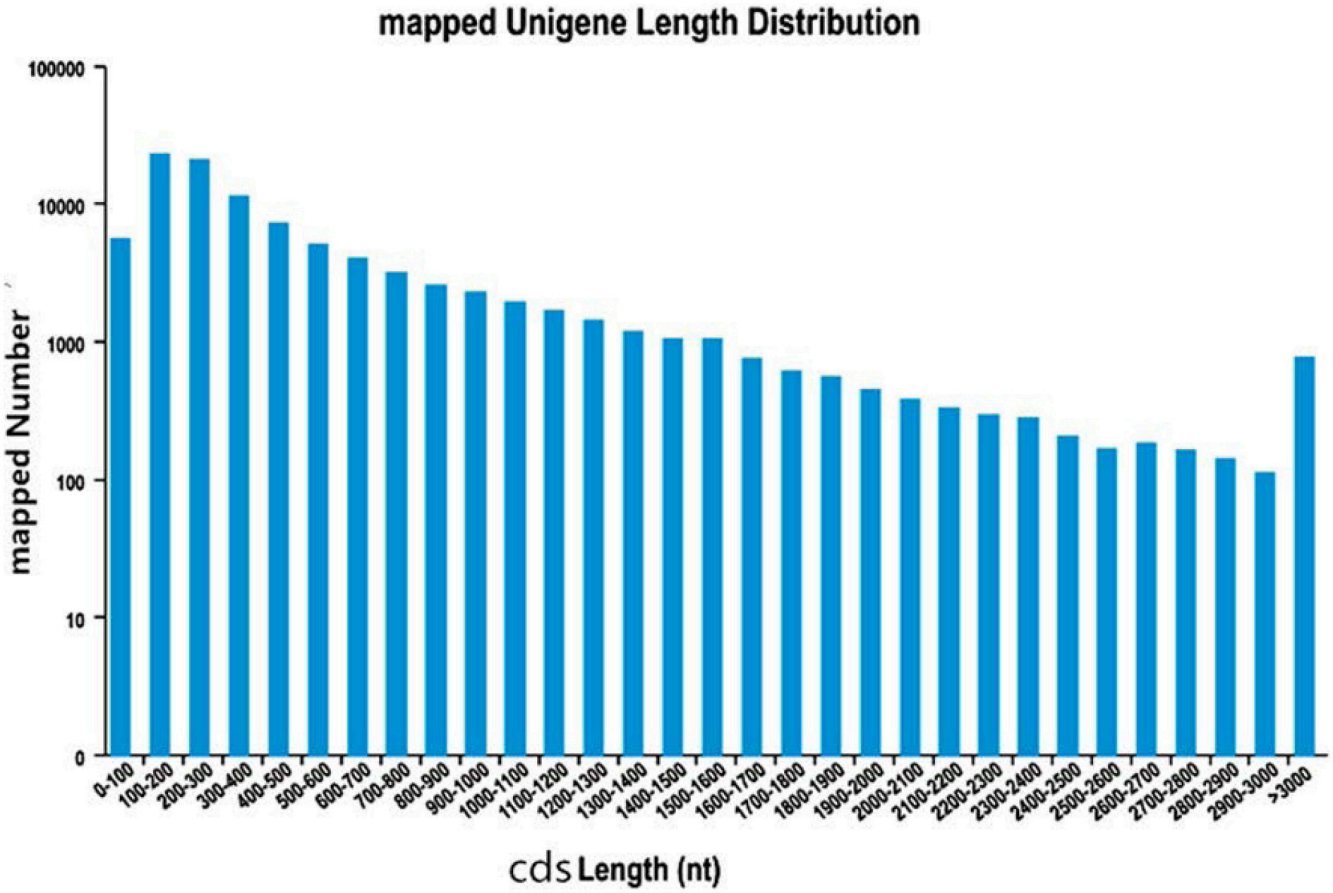
**The length distribution of CDSs mapped to know genes**.

**Figure 2:**
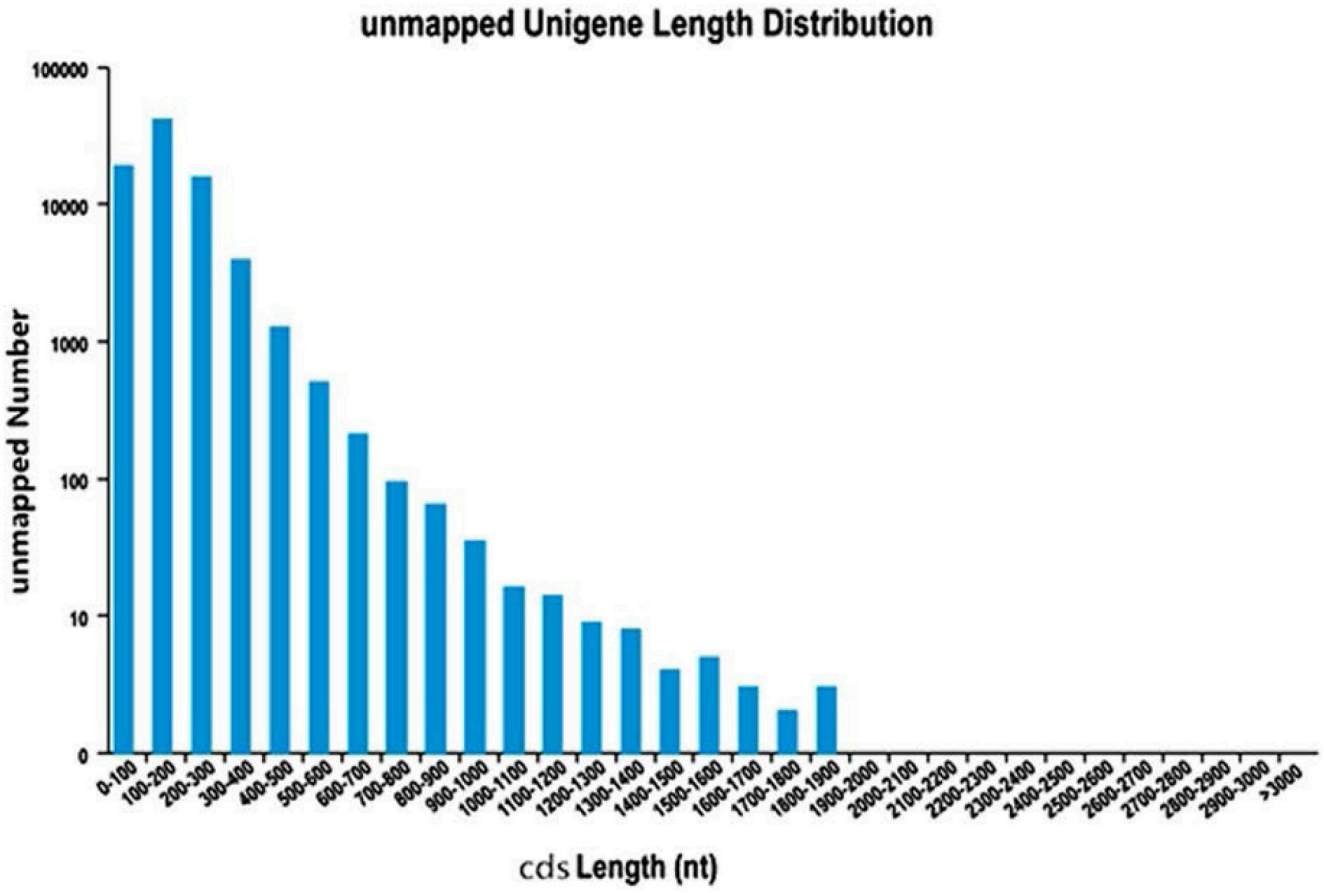
**The length distribution of CDSs unmapped to know genes**.

In order to predict CDSs, the unigenes for both “Yaan” and “1110” were consolidated into a single set of sequences. We predicted a total of 180,932 CDSs in our study, of which 99,037 CDSs aligned to the eight previously discussed databases. The largest number of CDSs (23,182, 23.41%) was 100–200 nt long (Figure 1). An additional 81,895 CDSs without BLAST hits were predicted by the Getorf software. Interestingly, 100–200 nt long CDSs were also the highest in number in this group (40,973 CDSs, 50.03%; Figure 2).

**Figure 3:**
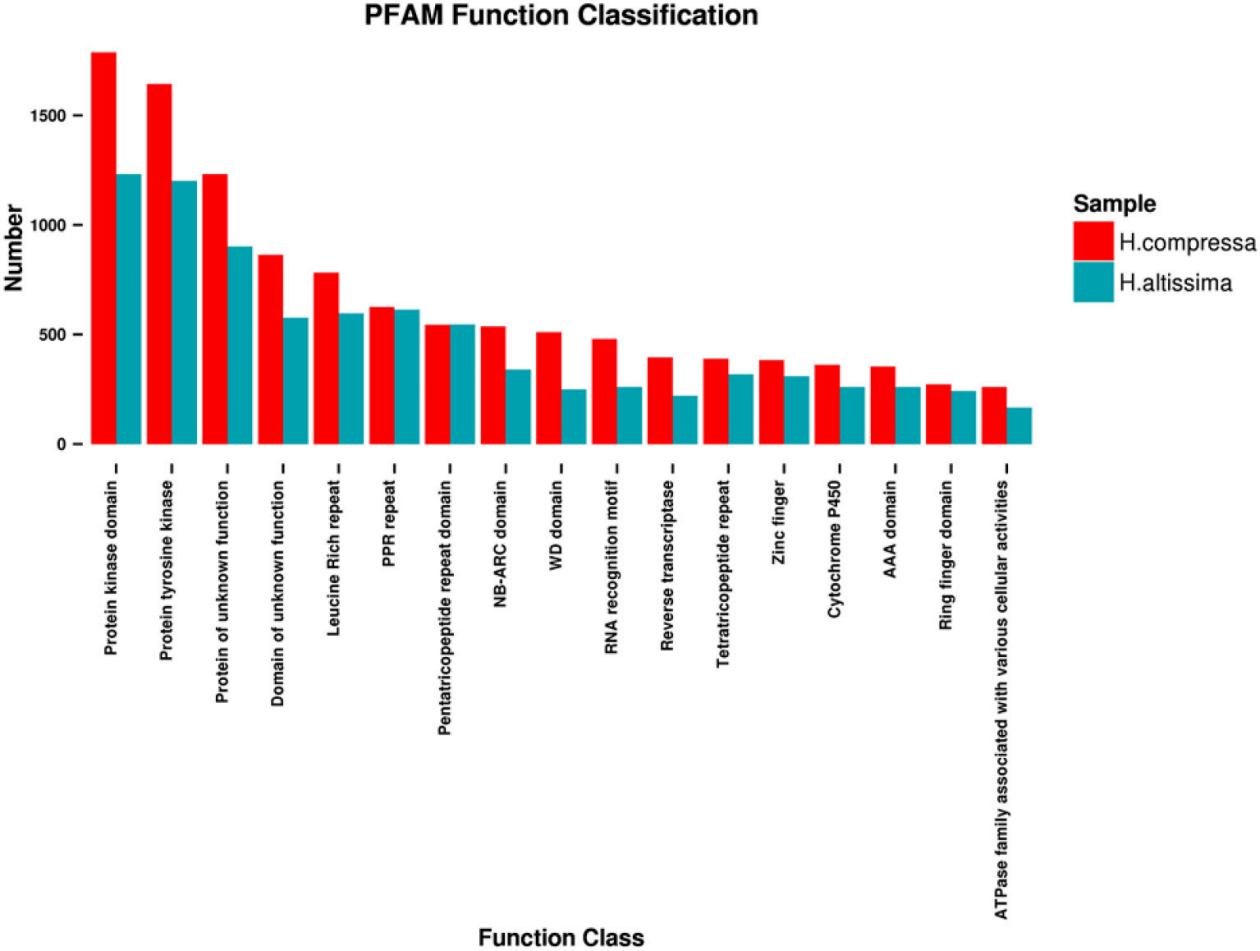
**Distribution of the 17 most abundant Pfam function classifications for *H. compressa* (“Yaan”) and *H. altissima* (“1110”)**.

The Pfam database is a comprehensive collection of protein domains and families, and its primary use in genome annotation is to quickly identify all genes that have the same protein domains (Bateman et al., 2004). A total of 44,285 and 25,190 unigenes of “Yaan” and “1110” were annotated to 4127 and 3584 Pfam protein families with an average of 10.73 and 7.03 unigenes per protein family, respectively. Of these identified protein families, the highest percentages of unigenes were predicted to contain a protein kinase domain (4.06% in “Yaan” and 4.89% in “1110”), a protein tyrosine kinase domain (3.71% in “Yaan” and 4.77% in “1110”), and protein domains with unknown functions (2.78% in “Yaan” and 3.58% in “1110”). Figure 3 shows the 17 most abundant Pfam protein families that were obtained from the two *Hemarthria* cultivars.

**Figure 4:**
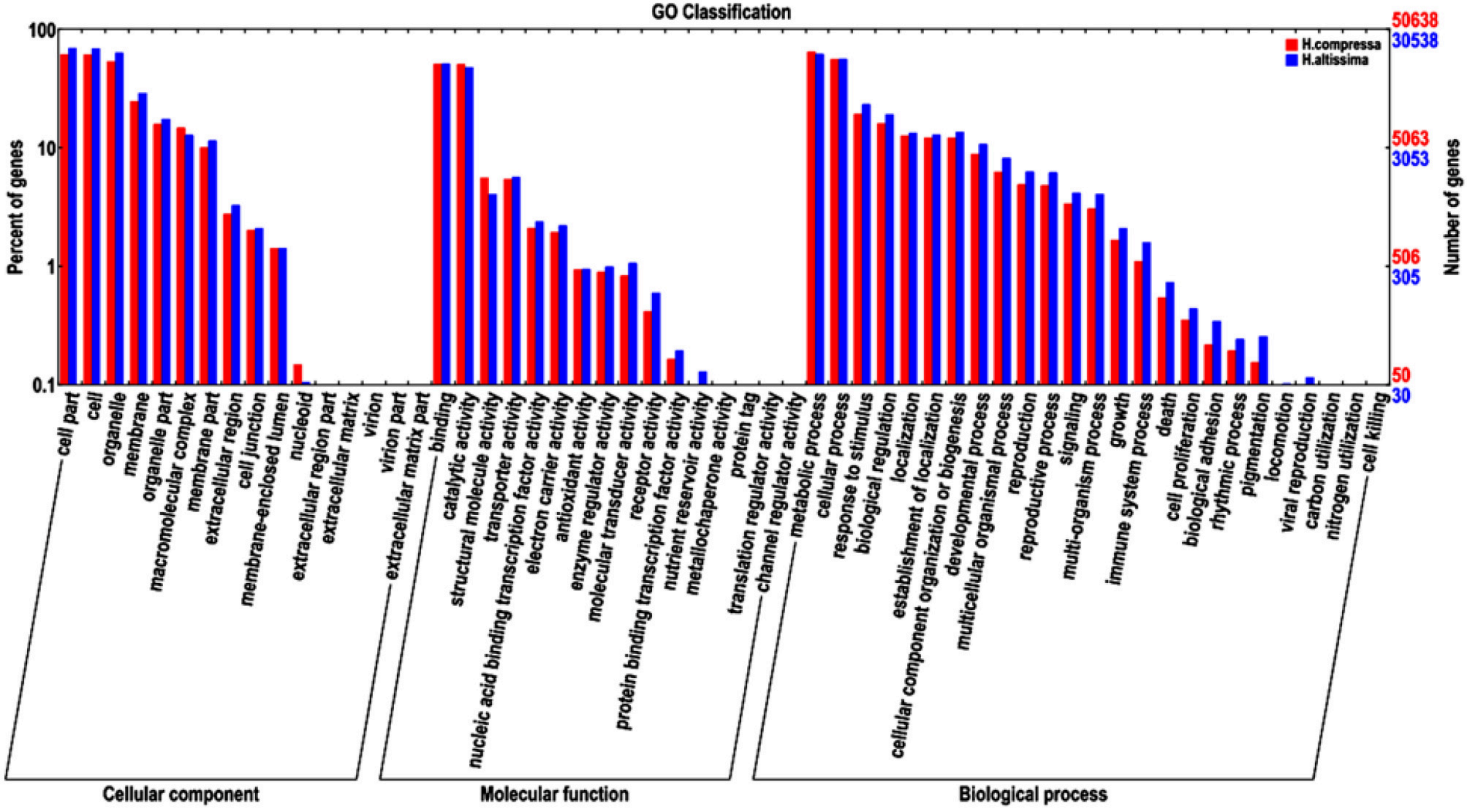
**Comparative distribution of GO categories for *H. compressa* (“Yaan”) and *H. altissima* (“1110”)**.

GO annotation is an international gene functional classification system that provides a controlled vocabulary that is able to comprehensively describe properties of the uncharacterized sequences in any organism (Gene Ontology Consortium, 2004; Tang et al., 2014). Based on sequence homology, 50,638 “Yaan” unigenes (36.92% of all the assembled “Yaan” unigenes) and 30,538 “1110” unigenes (39.58% of all the assembled “1110” unigenes) were assigned to at least one GO term and were classified into three groups: biological processes, molecular function, and cellular component. The distribution of GO groups for the two *Hemarthria* materials was extremely similar. As described in Figure 4, most of the unigenes were classified into the cellular components of cell part, cell, organelle, and membrane. In the molecular function group, binding, catalytic activity, structural molecules and transporter activity were found to be the four most frequent classes. Among 25 different biological process categories, the metabolic processes category was the most represented followed by cellular processes, response to stimuli, and biological regulation. With the help of GO functional classification, a large number of the unigenes were assigned to a wide range of biological processes, molecular functions, and cellular components. This information can provide a valuable resource for gene expression profile analysis, gene location, and gene isolation in *Hemarthria* species. In addition, the main GO classifications identified in this study pertained to fundamental biological processes. These results are similar to previously reported studies of *de novo* transcriptome analyses in the taproots of radish and the leaves of young tea plant (Wang et al., 2013; Wu et al., 2014).

**Figure 5:**
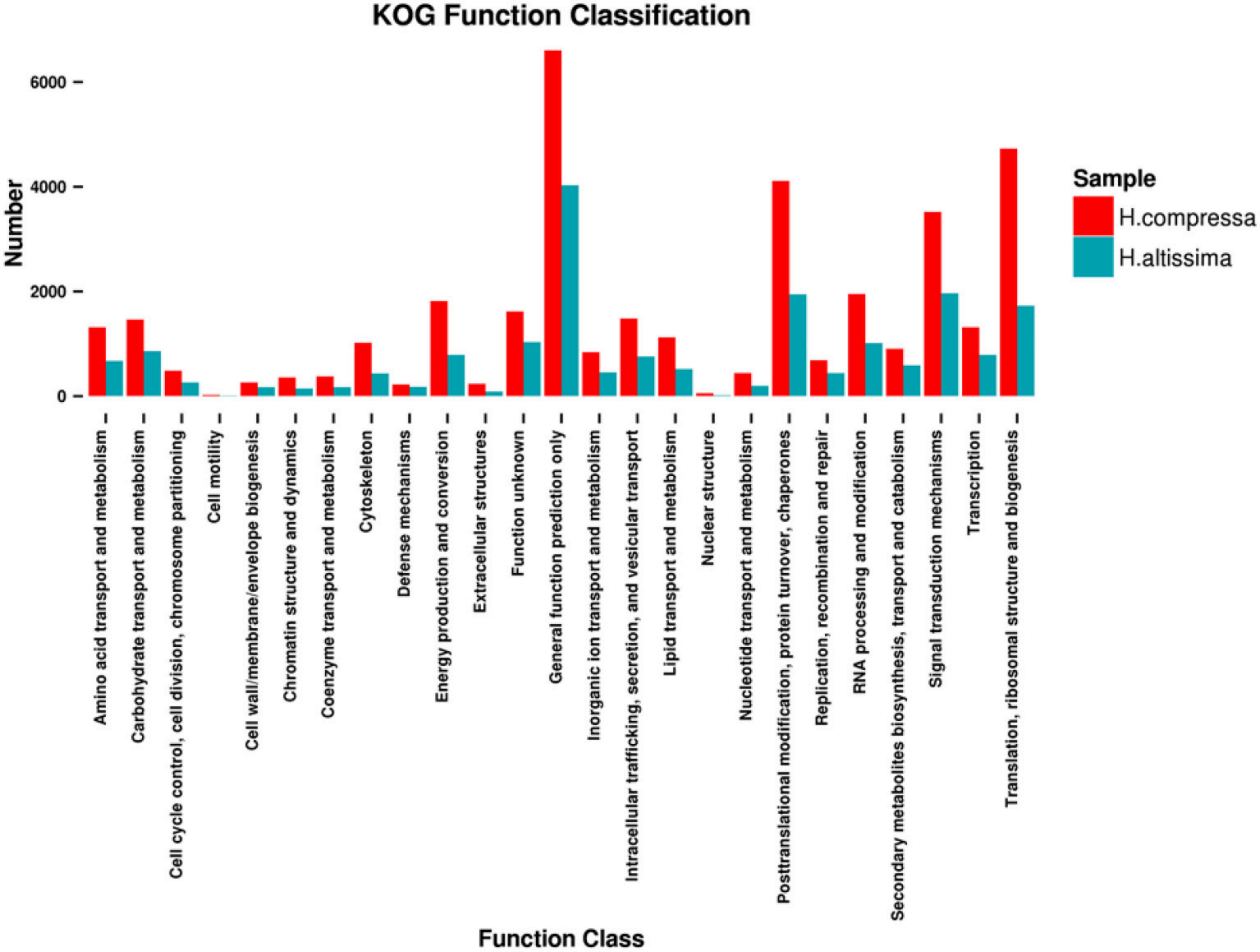
**Comparative distributions of KOG categories for *H. compressa* (“Yaan”) and *H. altissima* (“1110”)**.

For KOG analyses, 42,047 unigenes from “Yaan” were classified into 25 functional categories under four larger groups (metabolism, cellular processes and signaling, information storage and processing, and poorly characterized). Among these functional categories, the top three largest KOG categories were: 1) the prediction of general function only (6604 unigenes, 15.71%), 2) translation, ribosomal structure, and biogenesis (4730 unigenes, 11.25%), and 3) post-translational modification, protein turnover, and chaperones (4113 unigenes, 9.78%). Interestingly, only a few unigenes were assigned to extracellular structures (232 unigenes, 0.55%), nuclear structure (55 unigenes, 0.13%), and cell motility (20 unigenes, 0.05%). We identified several differences between the results from the KOG analyses for the “1110” unigenes and the “Yaan” unigenes. A total of 21,867 of the “1110” unigenes were distributed into 25 functional categories with the top three categories being: (1) general function prediction only (18.42%), (2) signal transduction mechanisms (8.99%), and (3) post-translational modification, protein turnover, and chaperones (8.88%). In both the “1110” and “Yaan” data, extracellular structures, nuclear structure, and cell motility categories had the smallest number of unigenes (Figure 5).

**Figure 6:**
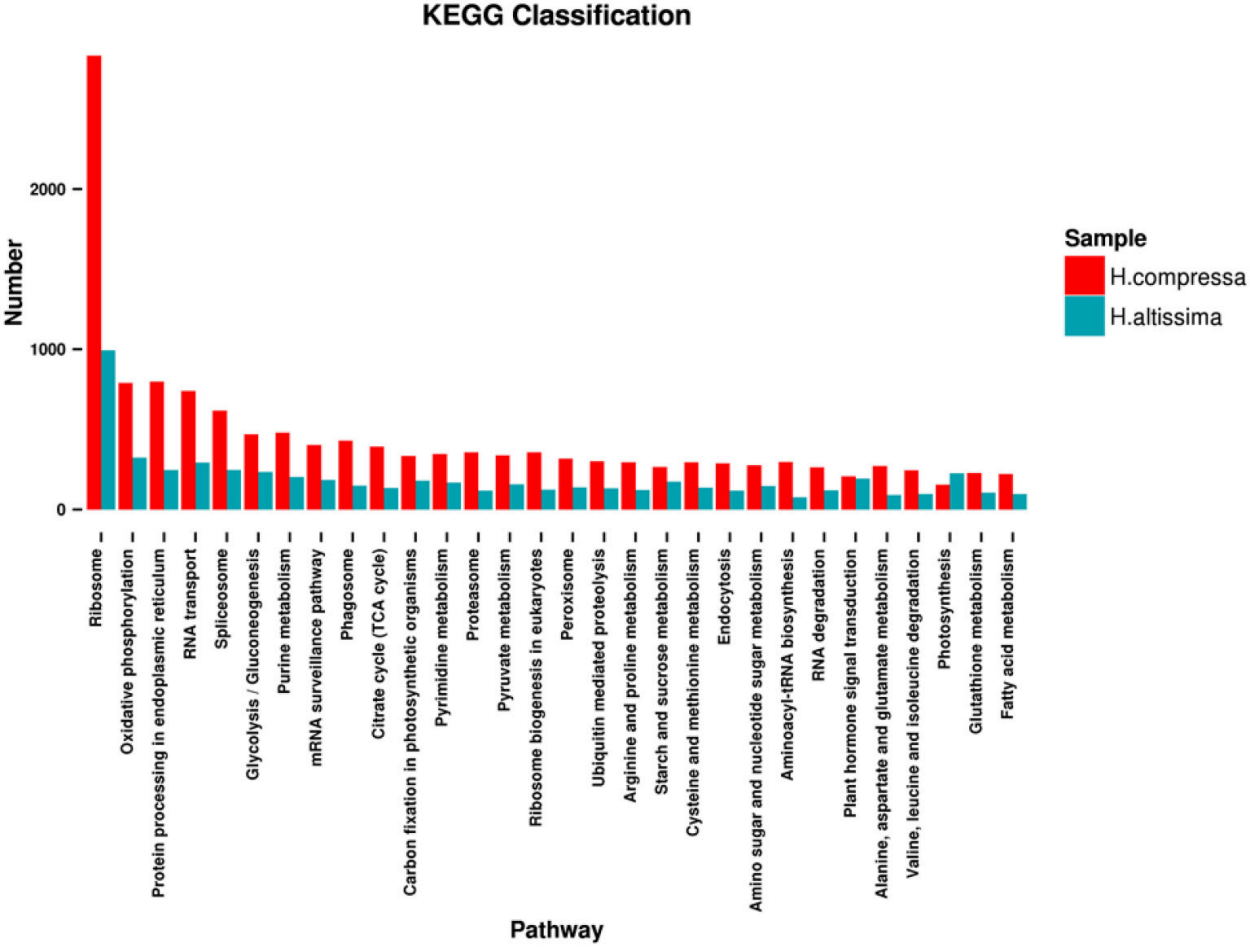
**KEGG classification of *H. compressa* (“Yaan”) unigenes and *H. altissima* (“1110”) unigenes**.

To systematically understand the biological pathways activated in *Hemarthria* roots and leaves, a KEGG analysis of the assembled unigenes of “Yaan” and “1110” was performed. A total of 18,148 and 8434 unigenes for “Yaan” and “1110,” respectively, were assigned to 121 biological pathways including transcription and translation, protein processing and modification, biosynthesis of secondary metabolites, signal transduction, replication and repair, transport and catabolism, immune system function, amino acid metabolism, and several others. The six major pathway groups for “Yaan” and “1110” were ribosomal [2832 (15.60%); 994 (11.79%)], oxidative phosphorylation [789 (4.35%); 324 (3.84%)], protein processing in the endoplasmic reticulum [798 (4.40%); 246 (2.92%)], RNA transport [739 (4.07%); 293 (3.47%)], spliceosomal [617 (3.40%); 247 (2.93%)], and glycolysis/gluconeogenesis [469 (2.58%); 234 (2.77%)] (Figure 6). These findings revealed a high level of protein synthesis occurring during the growth and development of *Hemarthria* species, and reflect the reliability of the KEGG orthology-based annotation. Furthermore, these data may provide reference information for future experiments such as gene expression analyses and gene cloning.

### Identification and validation of SNPs

Due to its high-throughput, accuracy, and reproducibility, RNA-Seq has become the preferred method of researchers to map and quantify transcriptomes (Vijay et al., 2013). RNA-Seq can be used to identify large numbers of genetic (heterozygous and homozygous) variants such as SNPs and SSRs, which can be further developed into molecular markers (Kaur et al., 2012). This is particularly useful in non-model species where a lack of genomic information hinders molecular marker identification and development. SNPs are the richest type of genetic variation distributed in eukaryotic genomes. They are widely considered the molecular marker of choice, especially in ecological and evolutionary studies, due to their potential for genome-wide coverage, high genotyping efficiency, data quality, automation, and analytical simplicity (Morin et al., 2004; Liu et al., 2011; Dou et al., 2012).

In this study, a total of 42,646 high-quality SNPs were detected in the 10,880 unigenes of the two *Hemarthria* genotypes, the majority of which (99.40%) were annotated. As summarized in Table 4, out of the 42,646 putative SNPs, the proportions of transversions were: 8.76% A/C; 6.45% A/T; 12.15% C/G, and 8.53% G/T. Additionally, the proportions of transitions were: 31.96% A/G and 32.15% C/T. The percentage of transition variations (64.11%) were approximately 1.79 times more frequent than transversions (35.89%) in these two *Hemarthria* genotypes. The prevalence of transitions found in these two cultivars was similar to those observed in other plants (Ebersberger et al., 2002; Renaut et al., 2010; Jhanwar et al., 2012).

**Table 4:** **Summary of putative SNPs identified from the two *Hemarthria* materials: *H. compressa* “Yaan” and *H. altissima* “1110”**.

**SNP information**	**Counts**
**TRANSVERSION**	
A/C	3734
A/T	2752
C/G	5183
G/T	3636
**TRANSITION**	
A/G	13,630
C/T	13,711
Total SNPs	42,646
Number of unigenes containing SNPs	10,880
Number of annotated unigenes containing SNPs	10,815
Number of SNPs in CDS	32,166
Number of SNPs in non-CDS	10,480
Number of SNPs in non-synonymous	12,707
Number of SNPs in synonymous	19,459

Further analysis of the SNPs revealed that 32,166 (75.43%) of the putative SNPs were distributed in sequences with CDSs and the remainder were in non-CDS sequences. The large number of SNPs located in sequences containing CDSs is possibly beneficial for future association with important economic and agronomic traits. These SNPs could be useful for molecular breeding programs and marker-assisted selection practices, as well as for understanding the phenotype differences between *Hemarthria* species (Huang et al., 2015). Additionally, 39.50% of the SNPs in the CDS regions resulted in non-synonymous substitutions, which corresponded to a change in the amino acid sequence of the translated peptide. These SNPs would be promising candidates for the study of mutations that may alter protein structure and function, and for an association analysis of *Hemarthria* germplasms.

Fifteen primer pairs were designed to validate putative SNPs that were identified in this study. We tested our primers on a diverse collection of 20 different *Hemarthria* clonal materials to determine if these SNPs could be used as molecular markers for other *Hermarthria* species (Table S2). After electrophoresis detection, we selected 10 out of 16 primer pairs that provided a clear, single band of the expected fragment size for sequencing. These 10 primer pairs were used to detect 37 SNPs; however, three of the primer pairs failed during sequencing. Twenty-nine (78.38%) of the 37 putative SNP loci were successfully validated by the remaining seven primer pairs. These SNPs were determined to be polymorphic with a range from two to eight loci per primer pair and an average of 4.1 loci (Table S3). These results suggest that the majority of the putative SNPs identified in this study are expected to be valid. Since the PCR was unable to detect some SNPs, some false SNPs may have been identified; however, we believe the false discovery rate is relatively low. The abundance of SNPs identified in this study will enhance the amount of useful and informative functional genetic marker resources that are available for *Hemarthria*, and these SNPs can readily be utilized for various maker-based applications in *Hemarthria* genetics, genomics, and breeding.

### Identification, validation, and cross-species transferability of SSRs

In plant genetics, SSRs are also widely considered to be molecular markers of choice due to their broad range of applications in processes such as MAS, genotype identification, genetic mapping, and the molecular tagging of genes. However, prior to this study, the paucity of available and robust SSRs had restricted the genetic analysis of this important genus. Therefore, there is an urgent need to develop a large set of SSRs for use in *Hermarthria* genetic studies and breeding programs.

Using a MISA perl script, all 182,842 unigenes from the two *Hemarthria* genotypes were searched for the presence of microsatellites. We identified a total of 8330 (4.56%) unigenes, 7,701 (92.45%) of which were annotated, that contained 10,888 SSRs with motif lengths ranging from one to six bp. Five hundred and seventy-five SSRs were present in compound types, meaning that they contained stretches of two or more different repeats. Data analysis of the SSR motifs revealed that the mono-nucleotide and tri-nucleotide repeats were the most abundant SSRs detected and accounted for 43.87 and 41.45%, respectively, of the total amount of SSRs. These were followed by the di-, tetra-, penta-, and hexa-nucleotide repeats, which accounted for 12.74, 1.43, 0.26, and 0.25% of the total number of SSRs, respectively (Table 5).

**Table 5:** **Summary of simple sequence repeats (SSRs) identified from the combined *Hemarthria* materials**.

**SSR information**	**Number**
Total number of sequences examined	182,842
Total number of identified SSRs	10,888
Number of sequences containing SSRs	8330
Number of known genes containing SSRs	7701
Number of sequences containing more than 1 SSR	1936
Number of SSRs present in compound formation	575
Mono-nucleotide repeats	4777
Di-nucleotide repeats	1387
Tri-nucleotide repeats	4513
Tetra-nucleotide repeats	156
Penta-nucleotide repeats	28
Hexa-nucleotide repeats	27

Due to a high probability of homopolymers existing as a quality problem associated with sequencing and genotyping errors, mono-nucleotides were excluded in the subsequent analysis (Gilles et al., 2011). In our study, tri-nucleotide repeat units were predominant, which is consistent with the results of similar studies in other plant genera (Jhanwar et al., 2012; Kaur et al., 2012; Yates et al., 2014). The prevalence of tri-nucleotide expansions within translated regions, in comparison to other nucleotide repeats, could be explained by slippage of the DNA replication machinery and the overall need to keep the integrity of the reading-frame.

The number of replications in a given repeat unit of SSRs ranged from five to >10, of which five reiterations were the most abundant. As the number of nucleotides in the microsatellite increased, the frequency of the given SSR structure (di-, tri-, tetra-, penta-, and hexa-nucleotide repeats) in our dataset progressively decreased (Table 6). This could be attributed to the relative probability of replication slippage events (Kaur et al., 2012). We also examined the repeat motif types of the SSR di- and tri-nucleotides. The results showed that the AG/TC motif had the largest frequency of up to 49.68% in the four types of di-nucleotide repeat motifs, and that the CCG/GGC motif was the most abundant in the tri-nucleotide SSRs, accounting for 48.19% (Table 7). Interestingly, the AG/TC of di-nucleotide SSRs was also found to be the most abundant motif in cereal crops (Temnykh et al., 2000; Kantety et al., 2002). A possible explanation for this is that the inverse GA/CT motif can represent multiple codons (such as GAG, AGA, UCU, and CUC) when transcribed into mRNA, and can translate into different amino acids (Arg, Glu, Ala, and Leu), among which Ala and Leu separately occupy a high proportion (8 and 10%) in proteins (Kantety et al., 2002). The most abundant tri-nucleotide motif in the genomes of other plants was also the inverse GGC/CCG motif (Nicot et al., 2004).

**Table 6:** **Summary information on frequencies of different SSR repeat motif types related to variation of repeat unit numbers in *Hemarthria* SSRs**.

**Motif length**	**Repeat unit number**								
	**5**	**6**	**7**	**8**	**9**	**10**	**>10**	**Total**	**%**
Di	–	591	317	181	129	94	75	1387	22.70
Tri	3079	1096	300	33	–	2	3	4513	73.85
Tetra	123	23	4	3	–	–	3	156	2.55
Penta	22	3	3	-	–	–	–	28	0.46
Hexa	14	6	5	1	1	–	–	27	0.44
Total	3238	1719	629	218	130	96	81	6111	–
%	52.99	28.13	10.30	3.57	2.13	1.57	1.32	–	–

**Table 7:** **Statistics of repeat motifs**.

**Motif length**	**Repeat motif**			
Di	AG/TC(49.68%)	AC/TG(22.93%)	AT/TA(15.14%)	CG/GC(12.26%)
Tri	CCG/GGC(48.19%)	AGC/TCG(14.45%)	AGG/TCC(11.30%)	ACG/TGC(7.93%)

For forage breeding, SSRs located in the CDS regions of the genome may be of more interest than SSRs that reside in non-CDS sequences because of the association of with important economic and agronomic traits. Among the 6111 SSRs (not including mono-nucleotide repeat SSRs) identified in this study, there were 1303 (21.32%) and 4808 (78.68%) SSRs identified in CDSs and non-CDSs sequences, respectively.

To validate the SSRs identified in this study, we successfully designed at least one primer pair for 4846 (79.30%) of the 6111 SSRs. A subset of 54 SSR primer pairs was selected for validation. Approximately 44 different *Hemarthria* clonal materials from different genetic backgrounds were utilized to test if these primers could be applied to other *Hemarthria* species (Table S2). The SSR primers were evaluated for their potential to amplify the target sequence and detect polymorphisms. In 34 (62.96%) cases, PCR products containing rich polymorphisms could be amplified from genomic DNA. The remaining 20 SSR primer pairs resulted in either weak or no amplification and were eliminated from further analysis. As seen in Table S4, these 34 SSR primer pairs covered di-, tri-, and tetra-nucleotide motifs and detected a total of 441 alleles. The number of PCR products for these primer pairs ranged from seven to 19 with an average of 12.97. Of the total number of alleles detected, 420 (95.24%) were determined to be polymorphic. The primer pairs for these alleles amplified six to 19 PCR products, with an average number of 12.35 PCR products per primer pair. The polymorphism information content (PIC) of the SSRs ranged from 0.5227 to 0.9496 with an equilibration of 0.7136, and the average Nei's gene diversity (H) and Shannon's information index of diversity (I) was calculated to be 0.2553 and 0.3991, respectively. These results revealed that the *Hemarthria* SSR markers developed in the current study have a high level of polymorphism, and that they may prove to be a valuable tool for population genetic studies, variety identification, association analysis, and MAS of *Hemarthria* species.

To further explore the transferability of SSR makers from *Hemarthria* to other Poaceae species, the 54 SSR primers used in the preceding part of the study were also employed to amplify SSRs from *M. sinensis* (awn), *D. glomerata* (orchardgrass), *Pennisetum*, and *L. multiflorum* (Italian rygrass). High levels of cross-species transferability were observed in all four species. The transferability from *Hemarthria* to *L. multiflorum* was the highest, at 87.04% (47/54), and to *Pennisetum, M. sinensis*, and *D. glomerata* at 61.11% (33/54), 83.33% (45/54), and 77.78% (42/54), respectively. In general, the ratio of transferability of the SSR markers roughly reflects the genetic relationships among the different species; a higher transferability indicates a closer genetic relationship (Zhang et al., 2014). In our study, the highest transferability was from *Hemarthria* to *L. multiflorum*, indicating that *Hemarthria* has a close genetic relationship with *L. multiflorum*, followed by *M. sinensis, D. glomerata*, and *Pennisetum*. These results are inconsistent with the taxonomic relationships of *Hemarthria* species, *M. sinensis*, and *Pennisetum* which belong to the same subfamily, while *L. multiflorum* and *D. glomerata* do not. It is possible that these relationships were seen because of the different amounts of materials used, which resulted in detecting varying degrees of nucleotide variation. In other words, the more materials from a species, the more nucleotide variation could be found. Additionally, the samples of each of the other Poaceae species may not accurately represent the corresponding species. Even so, transfer of the SSR markers could be utilized in other Poaceae species that also lack genomic resources.

## Conclusions

In this study, we performed Illumina RNA sequencing, as well as *de novo* transcriptome assembly and annotation, of two *Hemarthria* cultivars, *H. compressa* “Yaan” and *H. altissima* “1110.” Based on the newly assembled unigenes, we successfully identified, designed primers, and validated a large number of potential genetic SNP and SSR markers. A selected subset of these SNP and SSR primers were able to be applied to a number of genetically diverse *H. compressa* and *H. altissima* materials. Additionally, we were able to utilize the SSR primers designed for *Hemarthria* to detect SSRs in other Poaceae species, supporting the quality of the polymorphisms detected in this study and their transferability to other closely related species. The results of this study help us to gain a better understanding of *Hermarthria* genetics, and they can be directly used in future genomic studies aimed at improving this important forage crop.

## Data accessibility

Raw Illumina reads: NCBI SRA: SRP058845.

## Author contributions

XH performed experiments, analyzed data, and wrote the manuscript. DHY guided and edited the writing. KLH conceived the experiments, conducted the bioinformatics analysis, and guided the writing. KLH, JZ, and JDH organized the funding and collected the materials. DHY, JZ, LL, QXZ, WL, YP, XM, and HYY participated in one or more processes of materials cultivation, samples collection, data analysis and manuscript editing. All authors approved the final manuscript.

### Conflict of interest statement

The authors declare that the research was conducted in the absence of any commercial or financial relationships that could be construed as a potential conflict of interest.
