# Supplementary material for: De novo Transcriptome Analysis and Molecular Marker Development of Two Hemarthria Species
Source: Front Plant Sci. 2016 Apr 18;7:496. doi: 10.3389/fpls.2016.00496 (PMC4834353; doi:10.3389/fpls.2016.00496)
Supplement: Supplementary file 1 [file Table1.DOC]

Table S1 Characteristics of transcriptome sequencing for the four *Hemarthria* samples. Yaan cl: the mixed leaves of ‘Yaan’, Yaan cr: the mixed roots of ‘Yaan’, 1110 cl: the mixed leaves of ‘1110’, 1110 cr: the mixed roots of ‘1110’.

| **Samples** | **ID** | **No. of raw reads** | **Raw data (bp)** | **No. of clean reads** | **clean data (bp)** | | **GC (%)** | | **N (%)** | | **Q20%** | | **CycleQ20%** | | **Q30%** |
| --- | --- | --- | --- | --- | --- | --- | --- | --- | --- | --- | --- | --- | --- | --- | --- |
| Yaan cl | T01 | 27,233,997 | 5,501,267,394 | 27,011,918 | 5,453,391,438 | 55.41 | | 0.04 | | 92.45 | | 100.00 | | 86.24 | |
| Yaan cr | T02 | 25,295,298 | 5,109,650,196 | 25,105,343 | 5,069,394,016 | 54.62 | | 0.04 | | 91.98 | | 100.00 | | 85.66 | |
| 1110 cl | T03 | 24,528,821 | 4,954,821,842 | 24,240,921 | 4,893,578,986 | 54.64 | | 0.04 | | 92.66 | | 100.00 | | 86.49 | |
| 1110 cr | T04 | 26,513,426 | 5,355,712,052 | 26,308,747 | 5,312,280,803 | 55.69 | | 0.04 | | 91.91 | | 100.00 | | 85.47 | |
