# Supplementary material for: De novo Transcriptome Analysis and Molecular Marker Development of Two Hemarthria Species
Source: Front Plant Sci. 2016 Apr 18;7:496. doi: 10.3389/fpls.2016.00496 (PMC4834353; doi:10.3389/fpls.2016.00496)
Supplement: Supplementary file 2 [file Table2.DOC]

Table S2 Materials used for validation of marker assay performance. a: the materials used for validation of SSR marker; b: the materials were used to validate SNPs.

| **No.** | **Identity** | **Origin** | **Species** | **Plant types** | |
| --- | --- | --- | --- | --- | --- |
| 1 | H201^a,b^ | Zimbabwe, Africa | *Hemarthria altissima* | Wild materials | |
| 2 | H202^a,b^ | Transvaal, South Africa | *H. altissima* | Cultivar |  |
| 3 | H203^a,b^ | Limpopo, South Africa | *H. altissima* | Cultivar |  |
| 4 | H204^a,b^ | Transvaal, South Africa | *H. altissima* | Cultivar |  |
| 5 | H205^a^ | KwaZulu-Nata, South Africa | *H. altissima* | Wild |  |
| 6 | H206^a^ | Limpopo, South Africa | *H. altissima* | Wild |  |
| 7 | H207^a^ | Limpopo, South Africa | *H. altissima* | Wild |  |
| 8 | H208^a^ | Transvaal, South Africa | *H. altissima* | Wild |  |
| 9 | H210^a^ | KwaZulu-Nata, South Africa | *H. altissima* | Wild |  |
| 10 | H211^a,b^ | KwaZulu-Nata, South Africa | *H. altissima* | Wild |  |
| 11 | H212^a^ | KwaZulu-Nata, South Africa | *H. altissima* | Wild |  |
| 12 | H213^a^ | South Africa | *H. altissima* | Wild |  |
| 13 | H214^a^ | South Africa | *H. altissima* | Wild |  |
| 14 | H215^a,b^ | KwaZulu-Nata, South Africa | *H. altissima* | Wild |  |
| 15 | H217^a^ | KwaZulu-Nata, South Africa | *H. altissima* | Wild |  |
| 16 | H218^a^ | KwaZulu-Nata, South Africa | *H. altissima* | Wild |  |
| 17 | H221^a^ | KwaZulu-Nata, South Africa | *H. altissima* | Wild |  |
| 18 | H222^a^ | KwaZulu-Nata, South Africa | *H. altissima* | Wild |  |
| 19 | H224^a,b^ | South Africa | *H. altissima* | Wild |  |
| 20 | H225^a^ | Limpopo, South Africa | *H. altissima* | Wild |  |
| 21 | H226^a^ | Limpopo, South Africa | *H. altissima* | Wild |  |
| 22 | H227^a^ | Swaziland, Africa | *H. altissima* | Wild |  |
| 23 | H228^a,b^ | Zimbabwe, Africa | *H. altissima* | Wild |  |
| 24 | H229^a^ | Zimbabwe, Africa | *H. altissima* | Wild |  |
| 25 | H230^a^ | Transvaal, South Africa | *H. altissima* | Wild |  |
| 26 | H231^a^ | Limpopo, South Africa | *H. altissima* | Wild |  |
| 27 | H233^a^ | Limpopo, South Africa | *H. altissima* | Wild |  |
| 28 | H234^a^ | KwaZulu-Nata, South Africa | *H. altissima* | Wild |  |
| 29 | H235^a^ | KwaZulu-Nata, South Africa | *H. altissima* | Wild |  |
| 30 | H236^a^ | KwaZulu-Nata, South Africa | *H. altissima* | Wild |  |
| 31 | H237^a^ | Cape Provinc, South Africa | *H. altissima* | Wild |  |
| 32 | H238^a^ | Limpopo, South Africa | *H. altissima* | Wild |  |
| 33 | H239^a,b^ | Mauritius, East Africa | *H. altissima* | Wild |  |
| 34 | H240^a,b^ | Argentina , South America | *H. altissima* | Wild |  |
| 35 | H241^a,b^ | Japan | *H. compressa* | Wild |  |
| 36 | H242^a,b^ | New South Wales, Australia | *H. uncinata* | Wild |  |
| 37 | H244^a,b^ | Heilongjang, Meadow of the Songnen Plain | [*H. japonica*](http://202.115.182.57/kcms/detail/            search.aspx?dbcode=CJFQ&sfield=kw&skey=Hemarthria+japonica) | Wild |  |
| 38 | H021^a,b^ | Mianyang, Sichuan | *H. compressa* | Wild |  |
| 39 | H037^a,b^ | Daxian, Sichuan | *H. compressa* | Wild |  |
| 40 | H046^a,b^ | Libo, Guizhou | *H. compressa* | Wild |  |
| 41 | H051^a,b^ | Qiaojia, Yunnan | *H. compressa* | Wild |  |
| 42 | Guang Yi^a,b^ | Guangyi, Guangxi | *H. compressa* | Cultivar |  |
| 43 | Ya An^a,b^ | Ya’an, Sichuan | *H. compressa* | Cultivar |  |
| 44 | Chong Gao^a,b^ | Chongqing | *H. compressa* | Cultivar |  |
