# Supplementary material for: De novo Transcriptome Analysis and Molecular Marker Development of Two Hemarthria Species
Source: Front Plant Sci. 2016 Apr 18;7:496. doi: 10.3389/fpls.2016.00496 (PMC4834353; doi:10.3389/fpls.2016.00496)
Supplement: Supplementary file 3 [file Table3.DOC]

Table S3 Characterization of 29 novel SNP loci for 20 *Hemarthria* germplasm resources *A*=allele variation; *MAF*=minor allele frequence; h = Haplotype diversity; π = Nucleotide diversity

| **Contig name** | **Primer sequences (5'–3')** | **Location** | ***A*** | ***MAF*** | **h** | **π** | **Substitution type** |
| --- | --- | --- | --- | --- | --- | --- | --- |
| CL1162Contig1 | F: GGATTACGATGTTAGGTTACGTGAT | 2183 | G/T | 0.105 | 0.778 | 0.00976 | transversion |
|  | R: AAACAAAGACCACATTGTAGCTCAG | 2186 | C/T | 0.316 |  |  | transition |
|  |  | 2223 | A/G | 0.105 |  |  | transition |
|  |  | 2227  2229 | A/G  A/G | 0.158  0.421 |  |  | transition  transition |
| CL12Contig5 | F: AGTCCACTCCTATAGGTCACCAAAT | 5327 | A/G | 0.400 | 0.616 | 0.00277 | transition |
|  | R: AGAGACTAAATGGAAGGGACAGAAG | 5445 | A/G | 0.400 |  |  | transition |
| CL1436Contig2 | F: AGGAATACTGCTCTCTTCTGCTCTT | 593 | C/T | 0.105 | 0.877 | 0.00794 | transition |
|  | R: TCTTCTTCTGAACCAAACCATACTC | 709 | G/C | 0.368 |  |  | transversion |
|  |  | 714 | A/G | 0.421 |  |  | transition |
|  |  | 816 | C/T | 0.316 |  |  | transition |
| CL15998Contig1 | F: CAACTTCTGACTCCAGTACCAAACT | 503 | C/T | 0.100 | 0.800 | 0.00719 | transition |
|  | R: TGGATCCTGTTGTAGCAGAGATAAT | 571 | A/G | 0.300 |  |  | transition |
|  |  | 597 | C/T | 0.100 |  |  | transition |
| CL16022Contig1 | F: CGACATCTTCCAGTGGTATGTAACT | 426 | A/C | 0.100 | 0.658 0.00756 | | transversion |
|  | R: ATGGTGTAGGCCTTGTCAAACTTAT | 450 | C/T | 0.100 |  |  | transition |
|  |  | 471 | A/T | 0.100 |  |  | transversion |
|  |  | 489 | C/T | 0.100 |  |  | transition |
|  |  | 528 | A/T | 0.100 |  |  | transversion |
|  |  | 529  559 | A/G  A/C | 0.100  0.250 |  |  | transition |
|  |  |  |  |  |  |  | transversion |
|  |  | 581 | A/G | 0.350 |  |  | transition |
| CL16177Contig1 | F: TTACACACAACTGGCATAAGAACTG | 340 | C/T | 0.150 | 0.763 0.00881 | | transition |
|  | R: TGTAGCTGTAGAAATGTCGAAGATG | 354 | C/T | 0.150 |  |  | transition |
|  |  | 461 | C/T | 0.400 |  |  | transition |
| CL16204Contig1 | F: CGAAGTGGTTGCCATAGATGTACT | 518 | A/C | 0.150 | 0.795 | 0.00453 | transversion |
|  | R: CTTCCTCGACACCAACTCATTC | 722 | C/T | 0.400 |  |  | transition |
|  |  | 776 | C/T | 0.500 |  |  | transition |
|  |  | 867 | G/T | 0.100 |  |  | transversion |
