# Supplementary material for: De novo Transcriptome Analysis and Molecular Marker Development of Two Hemarthria Species
Source: Front Plant Sci. 2016 Apr 18;7:496. doi: 10.3389/fpls.2016.00496 (PMC4834353; doi:10.3389/fpls.2016.00496)
Supplement: Supplementary file 4 [file Table4.DOC]

Table S4 Characterisation of amplification patterns of 44 *Hemarthria* germplasm resources using 34 validated SSR primer pairs. Ta, annealing temperature of primer pair; NTA, number of total alleles; NPA, number of polymorphic alleles; PA, polymorphic alleles (%); PIC, polymorphic information content (%).

| **Primer code** | **Primer ID** | **Forward/Reverse primer (5'–3')** | **Repeat motif** | **Ta (°C)** | **Expected size (bp)** | **NTA** | **NPA** | **PA (%)** | **PIC (%)** |
| --- | --- | --- | --- | --- | --- | --- | --- | --- | --- |
| DHSSR1 | CL9Contig3 | F: GCCACAACCTTCACACCTTT | (CGC)6 | 52 | 313 | 10 | 9 | 90.00 | 0.8917 |
|  |  | R: CACCCAAGAAGGACAAGGAA |  |  |  |  |  |  |  |
| DHSSR2 | CL22Contig3 | F:TCACTCTGTGCGAGGAGTC | (CGC)5 | 52 | 297 | 16 | 16 | 100.00 | 0.8769 |
|  |  | R:ATGGACATCAACTAAGTCACC |  |  |  |  |  |  |  |
| DHSSR3 | CL54Contig1 | F:AACTCCTCTCCATCTGTTCTC | (GC)6 | 56 | 210 | 13 | 11 | 84.62 | 0.6652 |
|  |  | R:GAACCCTAGCTTTGCACAC |  |  |  |  |  |  |  |
| DHSSR4 | CL66Contig1 | F:GTAACTGGTTCTCCTGCTTCT | (TGC)5 | 56 | 256 | 11 | 11 | 100.00 | 0.5924 |
|  |  | R:ACTAGGTTACTGCTGCTTGTG |  |  |  |  |  |  |  |
| DHSSR5 | CL821Contig1 | F:ACGAGGACGAGAAGGAAG | (CGG)5 | 56 | 245 | 13 | 12 | 92.31 | 0.6559 |
|  |  | R:GCAGTACTTTTGCTACCAATG |  |  |  |  |  |  |  |
| DHSSR6 | CL965Contig1 | F:ATCACATACCTCGTGGACTC | (GGC)5 | 52 | 269 | 12 | 12 | 100.00 | 0.6999 |
|  |  | R:CTAGCAGACGCATCATCTATC |  |  |  |  |  |  |  |
| DHSSR7 | CL994Contig1 | F:AAGTACGGGTCCTCCTCTAC | (GGC)6 | 52 | 208 | 13 | 13 | 100.00 | 0.6668 |
|  |  | R:GAGAACCAGCTGAGCCTAAC |  |  |  |  |  |  |  |
| DHSSR8 | CL1079Contig1 | F:ACCTCATGGATCTCAAACAC | (GCG)6 | 52 | 235 | 14 | 14 | 100.00 | 0.8055 |
|  |  | R:ACTATGACGATGAGGACCAG |  |  |  |  |  |  |  |
| DHSSR11 | CL2144Contig1 | F:GAGCTTCTCCTTCTCCAACT | (GGC)5 | 56 | 264 | 16 | 15 | 93.75 | 0.6775 |
|  |  | R:ATCACCTTCGCTATCGTGT |  |  |  |  |  |  |  |
| DHSSR12 | CL2205Contig1 | F:AGTTTGATACACCTGCATGAC | (AC)7 | 56 | 150 | 11 | 11 | 100.00 | 0.7139 |
|  |  | R:GTAATCTTGTGTGTCCGTTTG |  |  |  |  |  |  |  |
| DHSSR14 | CL2526Contig1 | F:TAATGTAGCACTTGTCGAGGT | (CGC)5 | 56 | 274 | 8 | 7 | 87.50 | 0.7767 |
|  |  | R:CAGATGCTCTTCAAGACCTG |  |  |  |  |  |  |  |
| DHSSR15 | CL2597Contig1 | F:TCTTCGCCTTCACTTTCTC | (CGG)5 | 54 | 299 | 12 | 12 | 100.00 | 0.7954 |
|  |  | R:CATATCCATTCGGCTGAC |  |  |  |  |  |  |  |
| DHSSR16 | CL2812Contig1 | F:ATGCACTCCATAAAGATTCC | (GGC)5 | 54 | 251 | 12 | 10 | 83.33 | 0.5915 |
|  |  | R:AGCCTGTAAGGACTGAGGAT |  |  |  |  |  |  |  |
| DHSSR18 | CL3237Contig1 | F:GTAAGTTGGAACTTTGTCACC | (CGC)5 | 54 | 284 | 12 | 11 | 91.67 | 0.6106 |
|  |  | R:TTGTCCATCTTCTTGAGTCC |  |  |  |  |  |  |  |
| DHSSR19 | CL3531Contig1 | F:GGCTAAGAAGAGAAAGACAATG | (GCA)5 | 56 | 210 | 13 | 12 | 92.31 | 0.7135 |
|  |  | R:GACATGGTCAGATCCAAAGTA |  |  |  |  |  |  |  |
| DHSSR20 | CL3557Contig1 | F:CTGACTATCCAAAGCTCTCAA | (GGC)6 | 56 | 185 | 9 | 8 | 88.89 | 0.7881 |
|  |  | R:GATTATCCGCCTCTTTACCT |  |  |  |  |  |  |  |
| DHSSR25 | CL5320Contig1 | F:GGATCCCTCTCTTACTCCTTC | (GAG)5 | 56 | 191 | 18 | 18 | 100.00 | 0.7535 |
|  |  | R:AGAGGGAGAGAGGTCCATAG |  |  |  |  |  |  |  |
| DHSSR28 | CL5542Contig1 | F:GGTTTTGGTAAGATAGGAGGA | (AT)6 | 54 | 135 | 7 | 6 | 85.71 | 0.9496 |
|  |  | R:ACCAAGACCCTACAACAAACT |  |  |  |  |  |  |  |
| DHSSR31 | CL6065Contig1 | F:AGAGCTGGAGAAGAAGAGGA | (GCG)5 | 52 | 245 | 8 | 7 | 87.50 | 0.6302 |
|  |  | R:AGGAGGAGCTAGGGTTAGG |  |  |  |  |  |  |  |
| DHSSR32 | CL6226Contig1 | F:CAATGAAGATTTCCCAACAC | (CGC)6 | 52 | 276 | 10 | 9 | 90.00 | 0.6776 |
|  |  | R:TCCATGGTTGATGAGGTACTA |  |  |  |  |  |  |  |
| DHSSR34 | CL6426Contig1 | F:GGTGGTACTACCGCTACCTAT | (GCCG)5 | 54 | 268 | 19 | 19 | 100.00 | 0.8871 |
|  |  | R:AGTAACACAGCAGCAGAACAT |  |  |  |  |  |  |  |
| DHSSR40 | CL12069Contig1 | F:TCGAGGAAGAAGAGATGTAGA | (ATT)6 | 52 | 124 | 12 | 12 | 100.00 | 0.7809 |
|  |  | R:CTTTATTGGACAGCACCTTT |  |  |  |  |  |  |  |
| DHSSR43 | CL16122Contig1 | F:CGTTGAGGTCCACATCCT | (GCG)5 | 52 | 205 | 19 | 18 | 94.74 | 0.8923 |
|  |  | R:AACCCTTCTTTCCCGATG |  |  |  |  |  |  |  |
| DHSSR45 | CL16242Contig1 | F:ATCTTGTCCTTCTTGTCCTTG | (GCG)5 | 56 | 225 | 12 | 12 | 100.00 | 0.5227 |
|  |  | R:AAACCCTAGCTCTAGCAATTC |  |  |  |  |  |  |  |
| DHSSR46 | CL16253Contig1 | F:CTGGATCGTGTGGTGGTA | (CGG)5 | 52 | 137 | 14 | 14 | 100.00 | 0.8570 |
|  |  | R:CAGAACCCGAGAAAGTAAGAA |  |  |  |  |  |  |  |
| DHSSR47 | CL16265Contig1 | F:CAAGCACCTAGTAGACACGAC | (CGCA)5 | 56 | 227 | 13 | 13 | 100.00 | 0.6294 |
|  |  | R:GGTACTCGATGACCTTCTTG |  |  |  |  |  |  |  |
| DHSSR49 | CL17010Contig1 | F:GGGTACATCAAGGACATCATA | (GCAG)5 | 56 | 246 | 13 | 13 | 100.00 | 0.6594 |
|  |  | R:TAGCTCTGAATCCTCTCCTCT |  |  |  |  |  |  |  |
| DHSSR50 | CL17085Contig1 | F:CTCTTCCCTCTCTCCCTCT | (CGC)6 | 54 | 123 | 15 | 15 | 100.00 | 0.6765 |
|  |  | R:ATACTCCTCTCTGGCTACTCC |  |  |  |  |  |  |  |
| DHSSR51 | CL17142Contig1 | F:GTCGTCCTCCTTGAACAG | (TGG)5 | 54 | 257 | 10 | 10 | 100.00 | 0.5904 |
|  |  | R:ACTACTCCTCGCTCAACATCT |  |  |  |  |  |  |  |
| DHSSR52 | CL17301Contig1 | F:CACCTCAGTACACCTTGGAG | (CA)6 | 56 | 173 | 13 | 10 | 76.92 | 0.6311 |
|  |  | R:GTCCTCATCAGAGTCGTCAT |  |  |  |  |  |  |  |
| DHSSR54 | CL17615Contig1 | F:GACTCTTCATCCTCCTCCTC | (CGG)5 | 52 | 280 | 14 | 14 | 100.00 | 0.7000 |
|  |  | R:CGTTAATGCTCTCCTGGTTA |  |  |  |  |  |  |  |
| DHSSR56 | CL17649Contig1 | F:CGTGATTAGAGAAGGAGATCG | (GGC)5 | 56 | 166 | 13 | 12 | 92.31 | 0.5280 |
|  |  | R:GAGTTGACGGGTATGACG |  |  |  |  |  |  |  |
| DHSSR58 | CL18880Contig1 | F:CCGTACACCATCTCGTACA | (TGC)5 | 52 | 281 | 18 | 18 | 100.00 | 0.7708 |
|  |  | R:GTATTGTCGTGCCTGTTCA |  |  |  |  |  |  |  |
| DHSSR59 | CL18987Contig1 | F:GAGGCGGATGGATACTTAAC | (GCGG)5 | 52 | 227 | 18 | 16 | 88.89 | 0.6030 |
|  |  | R:GCAAAATCACATCTCGTCAC |  |  |  |  |  |  |  |
| Total | - | - | - | - | - | 441 | 420 | - | - |
| Average | - | - | - | - | - | 12.97 | 12.35 | 94.72 | 0.7136 |
