# Supplementary material for: De novo Transcriptome Analysis and Molecular Marker Development of Two Hemarthria Species
Source: Front Plant Sci. 2016 Apr 18;7:496. doi: 10.3389/fpls.2016.00496 (PMC4834353; doi:10.3389/fpls.2016.00496)
Supplement: Supplementary file 5 [file Table5.DOC]

Table S5 Materials used for exploring the transferability of SSR makers

| Species | individuals tested | Origin | Plant types |
| --- | --- | --- | --- |
| *Miscanthus sinensis* | 107 | Yanan, Sichuan | Wild materials |
|  | 814 | Luzhou, Sichuan | Wild materials |
|  | 1903 | Chongqing, China | Wild materials |
|  | 3608 | Guiyang, Guizhou | Wild materials |
| *Pennisetum* | Guiminyin | Guangxi, China | Cultivated variety |
|  | Ningza No.3 | Jiangshu, China | Cultivated variety |
|  | Rubrum | Yanan, Sichuan | Cultivated variety |
|  | Guimu No.1 | Guangxi, China | Cultivated variety |
|  | Huanan | Guangxi, China | Cultivated variety |
| *Dactylis glomerata* | Chuandong | Dazhou, Sichuan | Cultivated variety |
|  | Baoxing | Baoxing, Sichuan | Cultivated variety |
|  | Endurance | United States | Introduced variety |
|  | Baridana | Slovakia | Introduced variety |
|  | Grassland Wana | New Zealand | Introduced variety |
|  | Donata | Germany | Introduced variety |
|  | Sparta | United Kingdom | Introduced variety |
| *Lolium multiflorum* | Changjiang No.2 | Sichuan Agricultural University, Sichuan | Cultivated variety |
|  | Tetragold | DLF-Trifolium Group, Beijing | Introduced variety |
|  | Abundant | DLF-Trifolium Group, Beijing | Introduced variety |
|  | Aderenalin | Beijing Green Animal Husbandry S&T Development CO.,LTD., Beijing | Introduced variety |
|  | Double Barrel | Beijing Rytway Ecotechnology Co., LTD., Beijing | Introduced variety |
|  | Angus 1 | DLF-Trifolium Group, Beijing | Introduced variety |
